# Effect of Marine Reference on Inferred Evolutionary Patterns of Freshwater Stickleback

**DOI:** 10.1002/ece3.71461

**Published:** 2025-05-29

**Authors:** Brandon Tsai, Elizabeth Tapanes, Ainsley L. Fraser, Rana El‐Sabaawi, Diana J. Rennison

**Affiliations:** ^1^ Deparment of Ecology, Behavior & Evolution, School of Biological Sciences University of California San Diego La Jolla California USA; ^2^ Department of Biology University of Victoria Victoria British Columbia Canada

**Keywords:** evolution, fish, marine biology, parallelism, rapid adaptation

## Abstract

Threespine stickleback are a model system for studying rapid and parallel evolution. Studies characterizing freshwater evolution often use contemporary marines as an ancestral proxy, an approach that relies on untested assumptions about the lack of phenotypic variance in these marine fish. Here, we survey marine individuals collected from several sites, asking whether there is evidence of phenotypic variation. We identified considerable phenotypic variation among fish from different sites. Thus, we investigated the impact of this phenotypic variance on the inferred pattern of freshwater evolution. We tested whether estimates of the magnitude of phenotypic divergence or parallelism were affected by the choice of marine reference. We found that for freshwater populations, the magnitude of phenotypic divergence was dependent on marine sampling location—with divergence estimates differing by up to 65% with the substitution of marine reference site. Geographic distance and environmental similarity between marine and freshwater sites explained some of the variance in these divergence estimates. In contrast, across marine sites, neither geographic distance nor environmental similarity predicted morphological similarity, suggesting other factors drive morphological divergence among marine fish. The magnitude of phenotypic parallelism, estimated using a multivariate vector‐based approach, also differed significantly depending on the marine reference used. Together these results suggest that the choice of marine reference population, particularly its geographic distance from the focal population, is an important consideration when trying to characterize patterns of evolution in freshwater stickleback.

## Graphical abstract

We investigated the impact of marine phenotypic variance on the inferred pattern of freshwater evolution. For freshwater populations, the magnitude of phenotypic divergence and degree of parallel evolution were significantly dependent on marine sampling location.

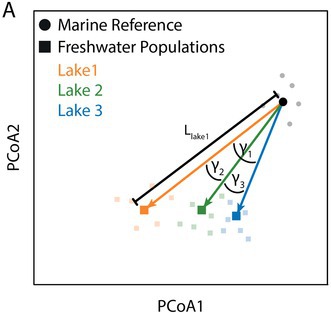

## 1 Introduction

Environmental conditions are changing rapidly due to anthropogenic disturbance, and rapid adaptive evolution will likely be crucial for the persistence of many taxa (Hoffmann and Sgrò 2011). Thus, many studies strive to understand the factors influencing rapid adaptation to new ecological conditions (Salamin et al. 2010). Patterns of divergence in response to differential ecological conditions depend on several key factors, including the strength of natural selection, amount of genetic variation, and population demography. Replicate populations adapted to similar ecological conditions provide a powerful opportunity to determine the relative contributions of deterministic and stochastic factors to adaptation (Schluter and Nagel 1995).

The threespine stickleback ( *Gasterosteus aculeatus* ) is a classic example of rapid evolution and divergence (Bell and Foster 1994), often used to explore the factors that affect adaptive capacity. After the last glacial maxima (around 12,000 years ago), anadromous marine threespine stickleback repeatedly and rapidly colonized freshwater habitats (Bell and Foster 1994). Today these post‐glacial populations exhibit multiple phenotypes thought to be adaptations to freshwater conditions (e.g., reduced salinity and predation; Lavin and Mcphail 1993; Colosimo et al. 2005; Deagle et al. 2012). For example, across these freshwater populations, increased body depth (Hendry et al. 2011), a reduction of lateral armor plating (Colosimo et al. 2004; Marchinko and Schluter 2007), and a reduction in gill raker number (Raeymaekers et al. 2007) are often found. These putatively adaptive phenotypes are often shared among independently derived freshwater populations (Cresko et al. 2004; Colosimo et al. 2005)—a pattern known as parallel evolution. Thus, this system provides a fantastic opportunity to assess factors promoting or constraining rapid adaptive evolution.

Unsurprisingly, many studies have leveraged the stickleback system to study patterns of rapid evolution (Bell et al. 2004; Barrett et al. 2011; Lescak et al. 2015; Rennison et al. 2016; Kingman et al. 2021). Conveniently, in this system, there are individuals that are found in the ancestral marine habitat. These contemporary marines can serve as proxies of the ancestral form when estimating the magnitude of freshwater evolution (Klepaker 1993; Jones et al. 2012; Pujolar et al. 2017). However, there are inherent assumptions of this approach, including that the contemporary marine populations are panmictic and phenotypically homogeneous, and that marine fish are largely unchanged from their ancestral founders. Of course, these assumptions are likely oversimplifications.

If assumptions of panmixia, phenotypic homogeneity and evolutionary stasis are correct, then choice of sampling location for a marine reference population would likely have little consequence on the inferred pattern of freshwater evolution. However, if these assumptions are to some degree violated, it could bias the biological inference with variable estimates of divergence. We know that over the past 12,000 years there have been rapid changes in many abiotic factors including ocean temperature and acidity (1988; Koenigstein et al. 2016; Durack et al. 2018; Burger et al. 2020). These changes in environmental factors have potentially exerted selective pressures on marine stickleback, perhaps heterogeneously. We also know that stickleback can adapt quickly—freshwater stickleback have been shown to exhibit heritable phenotypic change within a handful of generations (Bell et al. 2004; Kristjánsson 2005; Lescak et al. 2015). Thus, it is plausible that marine stickleback have also evolved on short timescales. Further, recent work suggests marine stickleback *can* display variation in key traits across populations and exhibit evidence of population structure (Morris et al. 2018), at least at larger geographic scales (i.e., the Pacific coast vs. Baltic Sea or California vs. Alaska; DeFaveri et al. 2013; DeFaveri and Merilä 2013; Jakubavičiūtė et al. 2018; Morris et al. 2018).

In this study, we sought to test the hypothesis of marine homogeneity and determine the effect of marine variation on estimates of divergence and parallelism among independently derived freshwater populations of stickleback. To test the hypothesis of marine stasis/invariance, we characterized patterns of phenotypic variation across marine and freshwater populations of stickleback collected in relatively close geographic proximity (~26–380 km apart). We predicted that marine stickleback would be phenotypically variable, but less so than freshwater populations, which are known to exhibit rapid ecological and morphological diversification (Bell and Foster 1994). To examine the effect of ‘ancestral’ marine reference choice on the inferred pattern of freshwater evolution we estimated the overall magnitude of phenotypic divergence between marine references and focal freshwater populations using two quantitative methods. To further examine the hypothesis of marine stasis we tested for the effects of geographic distance, ecology, and marine reference site on freshwater divergence. Prior work has shown that ecological similarity tends to decay with geographic distance, a product of increased differences of environmental conditions and the limitations of dispersal (Hubbell 2001; Soininen et al. 2007). Thus, we predicted that both geographically proximate and environmentally similar marine populations would yield more similar estimates of freshwater divergence. We then used a vector‐based approach to quantify the parallelism of freshwater evolution and tested whether marine reference affected the estimates.

## 2 Methods

### 2.1 Data Analysis

All data analysis was conducted using R (version 4.2.1; R Core Development Team 2023).

### 2.2 Phenotypic Variation

**FIGURE 1:**
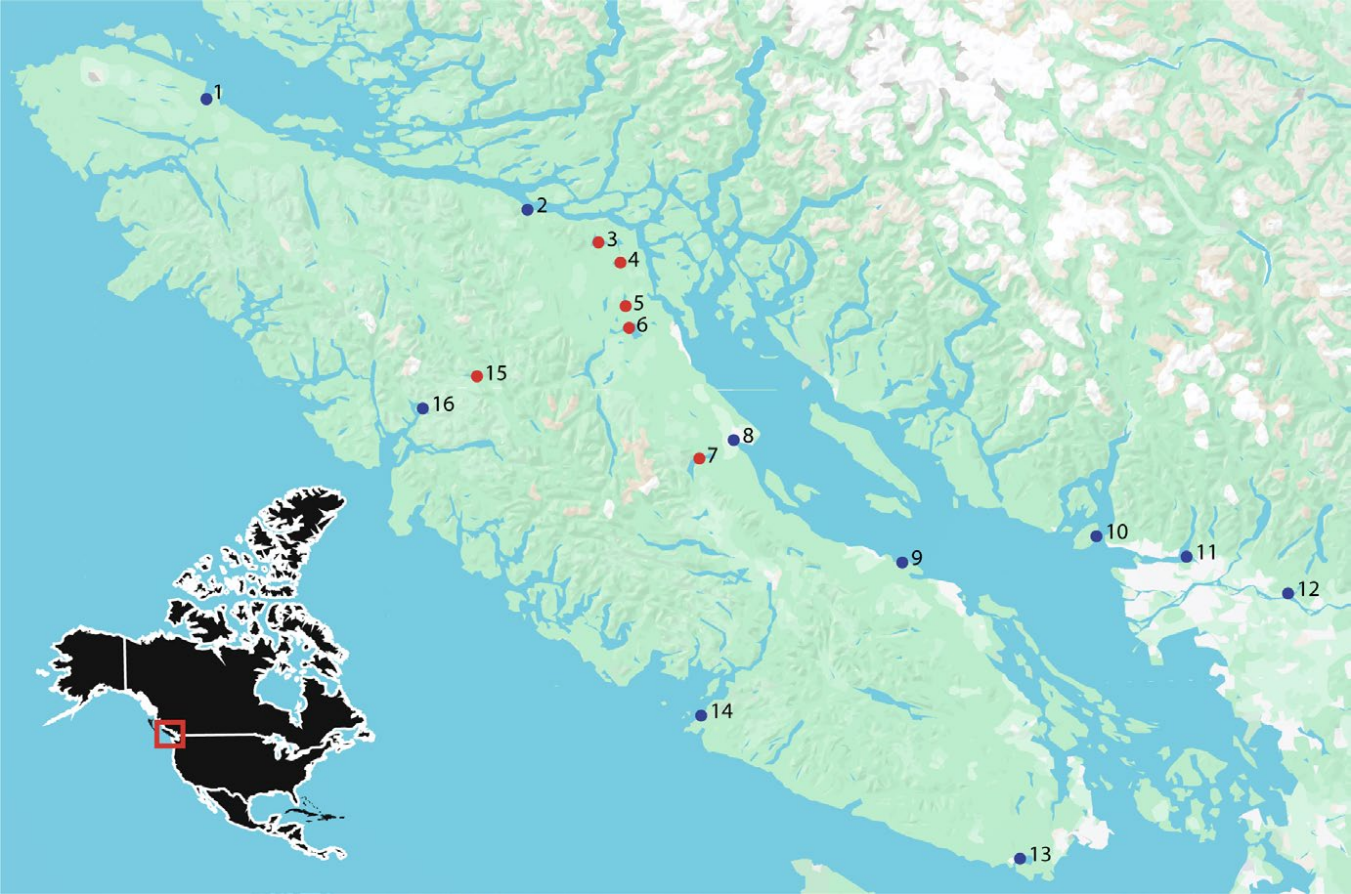
Sampling locations on Vancouver Island and the mainland of British Columbia, Canada. Region of interest on the west coast of North America is indicated by the red square on inset map. Freshwater populations are denoted as red circles and marine populations are denoted as blue circles. Numbers representing each sampling location are as follows: 1. Port Hardy Estuary, 2. Sayward Estuary; 3. Mccreight Lake; 4. Lil Mud Lake; 5. Mohun Lake; 6. Lower Campbell Lake; 7. Comox Lake; 8. Courtenay River Estuary; 9. Englishman River Estuary; 10. Bowen Lagoon; 11. Belcarra Inlet; 12. Kanaka Creek; 13. Sooke River; 14. Bamfield Inlet; 15. Muchalat Lake; 16. Canton Lagoon.

Threespine stickleback were collected between 2019 and 2022 from ten marine (*n* = 21–60 fish per site) and six freshwater (*n* = 27–60 fish per site) locations on Vancouver Island and the southwestern coast of mainland British Columbia, Canada (Figure 1, Table 1). Fish were caught using beach seines or unbaited minnow traps, euthanized, and stored in 100% ethanol.

**TABLE 1:** Sample size for each location.

Site name	Site type	Sample size
Mohun Lake	Freshwater	29
Muchalat Lake	Freshwater	25
Comox Lake	Freshwater	30
Kanaka Creek	Marine	20
Courtenay River Estuary	Marine	39
Canton Lagoon	Marine	40
Sayward Estuary	Marine	38
Sooke River	Marine	22
Bowen Lagoon	Marine	60
Belcarra Inlet	Marine	60
Englishman River Estuary	Marine	25
Port Hardy Estuary	Marine	20
Bamfield Inlet	Marine	27
Lil Mud Lake	Freshwater	30
Lower Campbell Lake	Freshwater	30
Mccreight Lake	Freshwater	29

**FIGURE 2:**
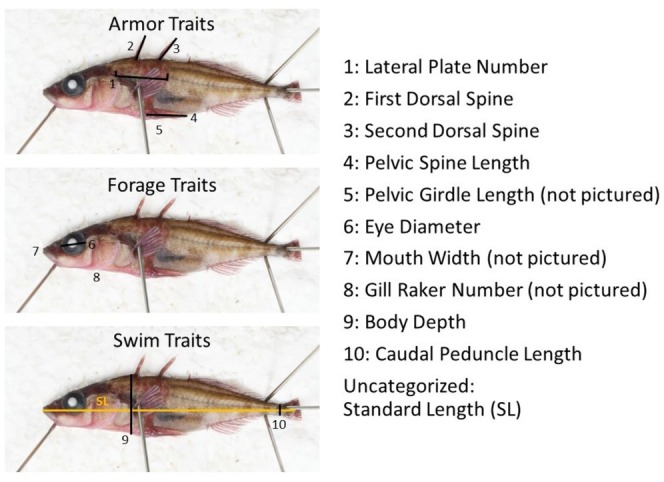
Morphological traits measured from stained stickleback specimens, categorized by function (armor, forage, and swim).

The ethanol preserved specimens were rehydrated, fixed with formalin, and stained with alizarin red to highlight bony structures (see Peichel et al. 2001 for full protocol details). From stained specimens, we estimated body depth, pelvic spine length, pelvic girdle length, first dorsal spine length, second dorsal spine length, mouth width, eye diameter, and caudal peduncle depth, using digital calipers (Figure 2). Lateral plate and gill raker number were manually counted. Each trait was measured three times, yielding intra‐ and inter‐scorer repeatability of scores within 5% of the average (range: 0.002%–3.5%).

Before subsequent analysis, all traits except lateral plate number in marine fish and gill raker number were size corrected (Bell 1981; Berner et al. 2010). Size corrections were performed separately for each trait and population, due to significantly different slopes. However, the use of a common slope for size correction did not result in a different interpretation of any downstream analyses, and the key patterns were unaltered (data not shown). Traits were size corrected based on the average standard length of the sample, using the following equation:

$${\mathrm{34𝛾}}_{\mathrm{34𝛾𝑖}}={\mathrm{34𝜒}}_{\mathrm{34𝜒𝑖}}-\mathrm{34𝛽}\left({\mathrm{34𝐿}}_{\mathrm{34𝐿𝑖}}-\mathrm{34𝐿}\right),$$

where 𝛾_𝑖_ is the size‐adjusted trait value, 𝜒_𝑖_ is the original trait value, 𝛽 is the regression coefficient ofunadjusted trait values on standard length, 𝐿_𝑖_ is the standard length of the individual and *L* is the average standard length of specimens in the dataset (50.71 mm). Levene's test in the *car* package (Fox and Weisberg 2019) was used to assess homogeneity of variance between the marine and freshwater samples.

### 2.3 Estimation of Phenotypic Divergence and Parallelism

To create a composite score for phenotypic divergence, we performed principal component analysis (PCA) using the morphological traits. PC1 described marine‐freshwater divergence with armor traits (lateral plating, pelvic spine & girdle, and dorsal spines) and body depth loading strongly on this axis. PC1 explained 43.3% of the variance. This first axis was then used to compute the absolute value of morphological divergence for each pairwise marine‐freshwater comparison. To determine the effect of marine reference on the magnitude of divergence, a general linear mixed‐effects model (GLMM) was used with freshwater population set as a random effect. PC2 explained 19.2% of the variance, with mouth width, eye diameter, and caudal peduncle length structuring variation along this axis. However, this axis did not strongly capture marine‐freshwater divergence; thus, we do not consider it in our analyses below. PC3 explained 13% of the morphological variance, with gill raker number and lateral plate number loading most strongly on this axis. Along PC3, we find differentiation between marine and freshwater sites; thus, we also used PC3 for some analyses below.

**FIGURE 3:**
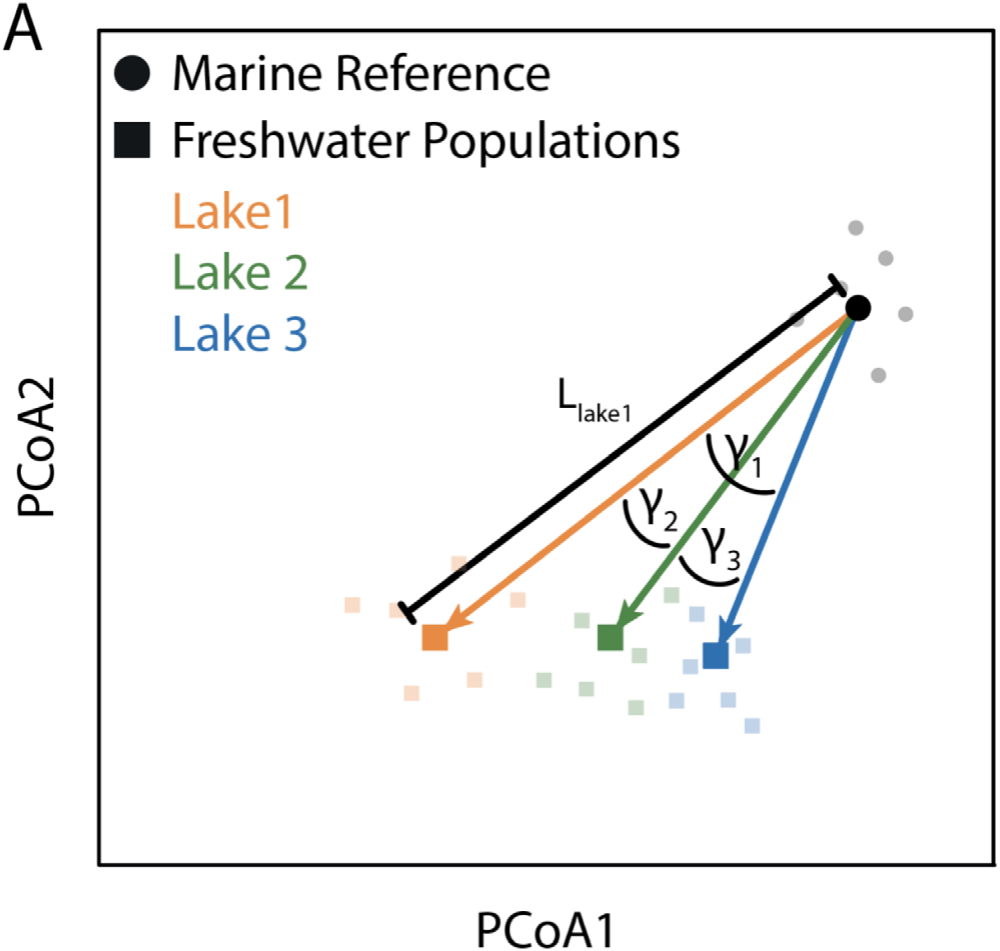
Illustration of multivariate vector analysis used for quantifying the magnitude and parallelism of freshwater morphological divergence of three hypothetical freshwater populations from a single hypothetical marine population using PCA loadings. Vectors (depicted in yellow, green and blue) connect the population means (centroids) of a single marine reference population and the three focal freshwater populations. For each vector connecting the marine and freshwater population the magnitude of divergence, L, can be estimated. Angles between marine‐freshwater vectors (γ) provide a quantitative measure of parallelism, indicating the similarity of evolutionary trajectories in multi‐dimensional space for each pair of vectors.

A multivariate vector‐based approach (Adams and Collyer 2009; Stuart et al. 2017; Härer and Rennison 2022) was used to estimate both the overall magnitude of phenotypic divergence between marine and freshwater populations and the degree of parallelism between pairs of marine and freshwater populations. Pairwise multivariate vectors were drawn between marine and freshwater pairs using eigenvalues from the PCA (illustrated in two dimensions in Figure 3). From these vectors, there were two metrics of interest: the length of the vector (an alternative approach to estimate the total magnitude of phenotypic divergence between freshwater and marine populations) and the angle (*theta*) between pairs of marine‐freshwater vectors (both metrics are depicted in Figure 3). Theta reflects the degree of parallelism between freshwater populations diverging from a single outgroup marine population. Angles below 90° are thought to be evidence of parallelism (Stuart et al. 2017). To estimate theta, pairwise comparisons were made for all marine‐freshwater vectors with the same marine reference population. For example, theta would be estimated between the Comox Lake—Bamfield Inlet vector and the Muchalat Lake—Bamfield Inlet vector. This process was repeated for each of the 10 marine references.

For estimation of divergence or parallelism using the panmictic reference population, the analyses were conducted as described above, except that all marine samples were coded as originating from the same site.

### 2.4 Environmental Variation

Geographic distance was estimated between sample sites using the shortest distance via waterways using a custom R script. The *sf* and *tmap* R packages were used to create the preliminary map (Kumar 1988; Pebesma 2018; Tennekes 2018) and rasterization was done using the *raster* and *fasterize* packages (Hijmans and van Etten 2021; Ross 2020).

To characterize one aspect of ecological similarity, we retrieved 19 bioclimatic variables from WorldClim for each population site using the *sp* and *raster* packages in R (Pebesma and Bivand 2005; Hijmans and van Etten 2021). A correlation matrix was estimated for the bioclimatic variables; in cases where variables were strongly correlated (*r* > 0.80) one of the variables was dropped. Among the correlated variables, we elected to retain the variables that described annual trends or variation, rather than monthly or quarterly data. Six bioclimatic variables were retained for further analysis: Annual mean temperature (BIO 01), Mean Diurnal Range (BIO 02), Isothermality (BIO 03), Mean Temperature of Wettest Quarter (BIO 08), Annual Precipitation (BIO 12) and Precipitation Seasonality (BIO 15).

We performed PCA on the six retained climate variables, and eigenvalues for climatic PCAs 1, 2, and 3 were extracted. To quantify differentiation in climate between sites, the absolute difference in eigenvalues was estimated for each pairwise marine‐freshwater comparison. In the environmental analysis, PC axes 1 and 2 accounted for ~66% of the total variance in climate, and the first 3 PCs cumulatively explained ~90% of the variance. PC1 primarily distinguished populations based on precipitation and diurnal temperature (BIO 12, BIO 02). PCs 2 and 3 had strong loading of precipitation, seasonality (BIO 08, 15) and isothermality (BIO 3) variables, respectively.

To determine the effects of environmental and geographic distance on estimates of phenotypic divergence, using the ‘lme4’ package in R (Bates et al. 2015) we ran a general linear mixed‐effects model (GLMM) between the morphological PCA based divergence estimate and either geographic distance or the environmental differences. Freshwater site (i.e., lake) was set as a random effect in all models, except in the case of marine only models. In the first model, we tested for a relationship between phenotypic divergence and geographic distance between marine and freshwater sites. In the second model, we tested for a relationship between phenotypic divergence and climatic difference.

## 3 Results

### 3.1 Patterns of Phenotypic Variation

**FIGURE 4:**
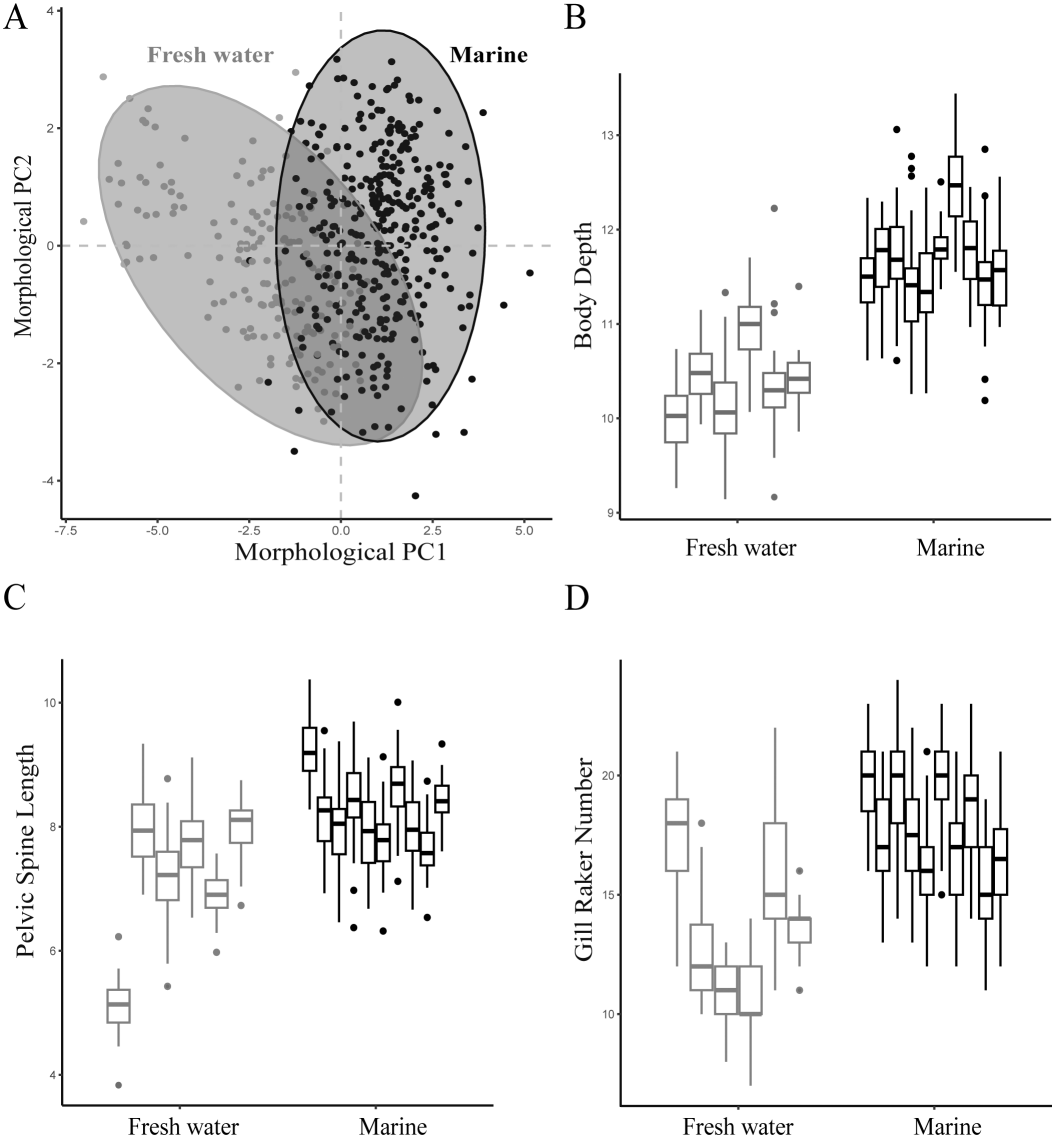
Morphological variation between marine and freshwater stickleback. PCA biplots of (A) marine and freshwater populations. Boxplots comparing variation in individual traits explaining divergence in PC1 between marine and freshwater populations: (B) Body depth (mm), (C) pelvic spine length (mm), and (D) gill raker number.

In the morphological PCA, Axis 1 accounted for 43% of the total variance in the dataset, with marine and freshwater populations clearly separated along this axis (Figure 4A). For several divergent traits, the amount of within‐population variation was found to be similar between marine and freshwater habitats (Table 2). For example, for body depth (Figure 4B) there was no difference in the variance among marine (*σ* ^2^ = 0.26) and freshwater (*σ* ^2^ = 0.26) samples. For other traits such as gill raker count and pelvic spine length, there was a significant difference in the variance between the marine and freshwater groups (gill raker *σ*_1_^2^–*σ*_2_^2^ = 3.7, *F*_1_ = 298.6, *p* < 0.001; pelvic spine *σ*_1_^2^–*σ*_2_^2^ = 1.1, *F*_1_ = 41.0, *p* < 0.001) (Figure 4C,D), with more variance seen among freshwater populations than marine for these traits. However, for several traits, marine populations are more variable than freshwater populations (Table 2) and marine populations exhibit variation in all traits, with the exception of lateral plate number where *σ* ^2^ = 0.02 (Table 2). When we look at PC1 eigenvalues, we find more across‐population variation in freshwater; specifically, there is 2.4 times more variation across freshwater populations than marine populations (*σ* ^2^ = 3.2 and 1.3 respectively).

**TABLE 2:** Results of Levene's test for homogeneity of variance between marine and freshwater samples.

Trait	Freshwater variance	Marine variance	*F*_1,528_	*p*
Body depth	0.26	0.26	0.01	0.906
**Gill raker number**	**6.92**	**10.64**	**7.61**	**0.006**
**Lateral plate number**	**1.02**	**0.02**	**298.62**	**> 0.0001**
**First dorsal spine length**	**0.51**	**0.61**	**6.54**	**0.011**
**Second dorsal spine length**	**0.55**	**0.32**	**15.71**	**> 0.001**
Mouth width	0.09	0.11	2.05	0.153
Eye diameter	0.08	0.06	3.78	0.052
**Caudal peduncle length**	**0.01**	**0.02**	**13.77**	**0.0002**
**Pelvic girdle length**	**1.76**	**0.61**	**46.5**	**> 0.0001**
**Pelvic spine length**	**1.33**	**0.26**	**41.03**	**> 0.0001**

### 3.2 Estimates of Phenotypic Divergence and Parallelism

**FIGURE 5:**
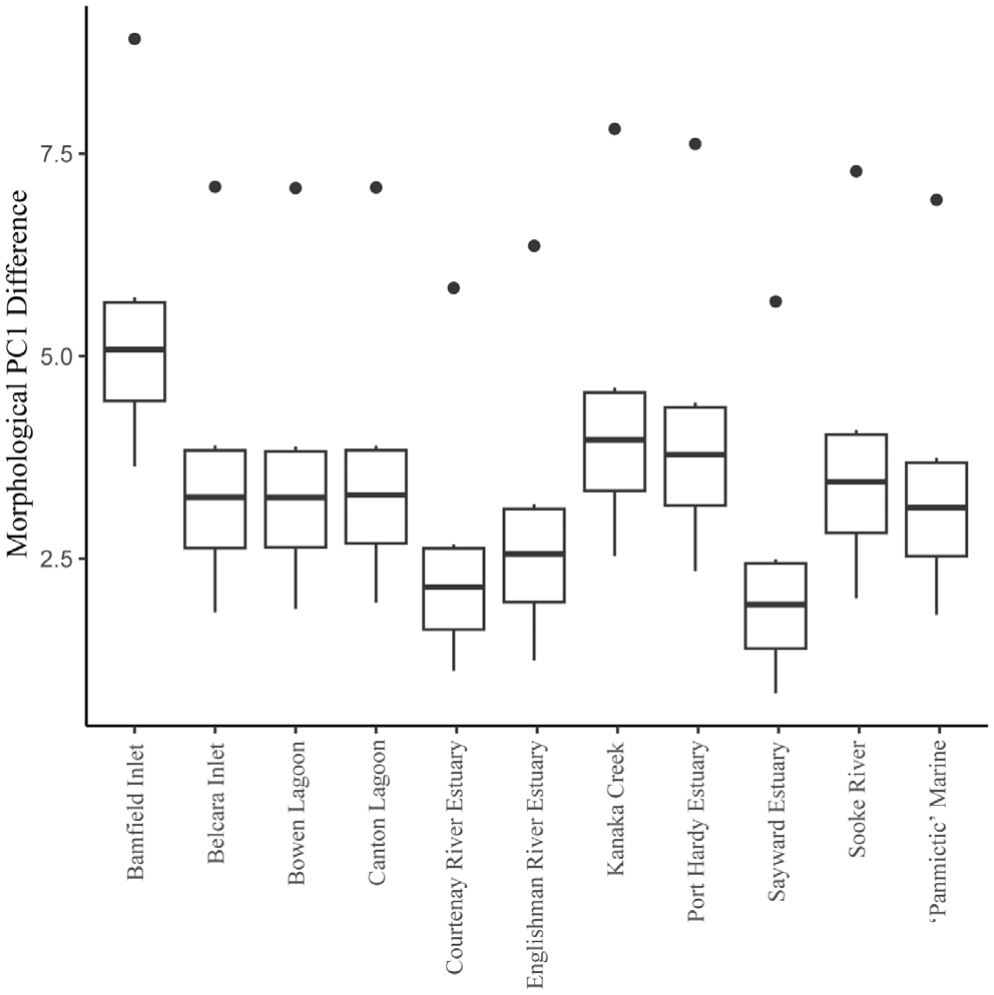
Effect of marine reference on estimates of morphological divergence for the six focal freshwater populations. Ten independent marine sampling locations are given along the *x*‐axis. Estimation of freshwater divergence is also shown using a ‘panmictic’ marine reference where marine samples have been pooled across all sites. Points plotted outside of the interquartile range and whisker, reflect outliers.

Estimates of the magnitude of freshwater divergence differed considerably across independent lake populations, and depended on the marine reference used (Figure 5). The magnitude of divergence, based on PC1 differences, differed significantly depending on marine reference population (Marine SS = 35.78, *F*_9_ = 883.38, *p* < 0.001); across marine references the average divergence ranged from 2.2 to 5.0, a difference of up to 2.2×. Depending on the focal freshwater population, use of different marine reference sites resulted in a 24%–65% change in the estimated magnitude of divergence. Correspondingly, the variance for freshwater site (random effect) was 2.9 (SE 1.7). When PC3 differences were used to describe marine‐freshwater differentiation the magnitude of divergence was typically less, ranging from 1.23 to 2.27. Yet, it was still the case that marine reference significantly affected the estimated magnitude of freshwater divergence (Marine SS = 6.7, *F*_9_ = 7.15, *p* < 0.001). We find that use of a ‘panmictic’ marine reference, where samples are pooled across all marine sites, resulted in intermediate estimates of freshwater divergence (Figure 5).

**FIGURE 6:**
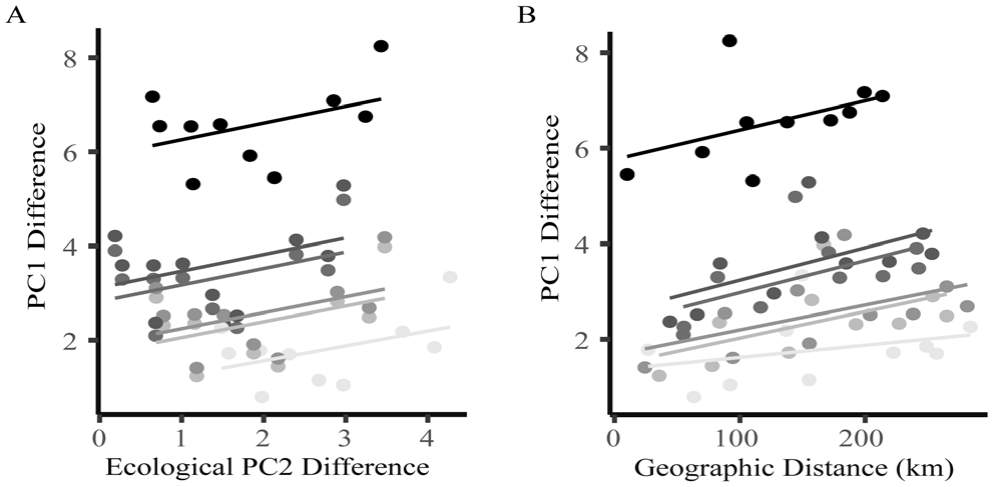
Association between the magnitude of morphological divergence (PC1 difference) and geographic distance or environmental difference. Relationship between morphological divergence (PC1 difference) with (A) ecological PC2 difference or (B) geographical distance. Each point on the scatterplot represents a pairwise comparison between a freshwater population and a marine population. Greyscale shade indicates different freshwater populations.

The magnitude of freshwater phenotypic divergence was significantly dependent on the geographic distance between the marine reference and focal freshwater site (*β* = 0.62, SE = 0.13, *t*_1_ = 4.65, *p* < 0.001). The variance for freshwater site (random effect) was 2.5 (SE 1.6), indicating considerable variation among lakes. However, for all freshwater populations, the magnitude of morphological divergence from marine reference populations scaled linearly and positively with geographic distance (Figure 6B). When environmental factors (PC2) were considered, the magnitude of morphological divergence had a significant positive linear relationship with environmental difference between marine and freshwater sites, which primarily represented aspects of climatic seasonality (*β* = 0.34, SE = 0.10, *t*_1_ = 3.52, *p* < 0.001; Figure 6B). Again, the variance for freshwater site (random effect) indicated considerable variation among lakes was 2.7 (SE 1.6). In contrast, the magnitude of morphological divergence among marine populations was not explained by geographic distance (*β* = 0.02, SE = 0.17, *t*_1,42_ = 0.11, *p* = 0.92) or ecological similarity (Climatic PC1: *β* = 0.04, SE = 0.07, *t*_1,42_ = 0.65, *p* = 0.52; Climatic PC2: *β* = 0.14, SE = 0.13, *t*_1,42_ = 1.07, *p* = 0.29).

**FIGURE 7:**
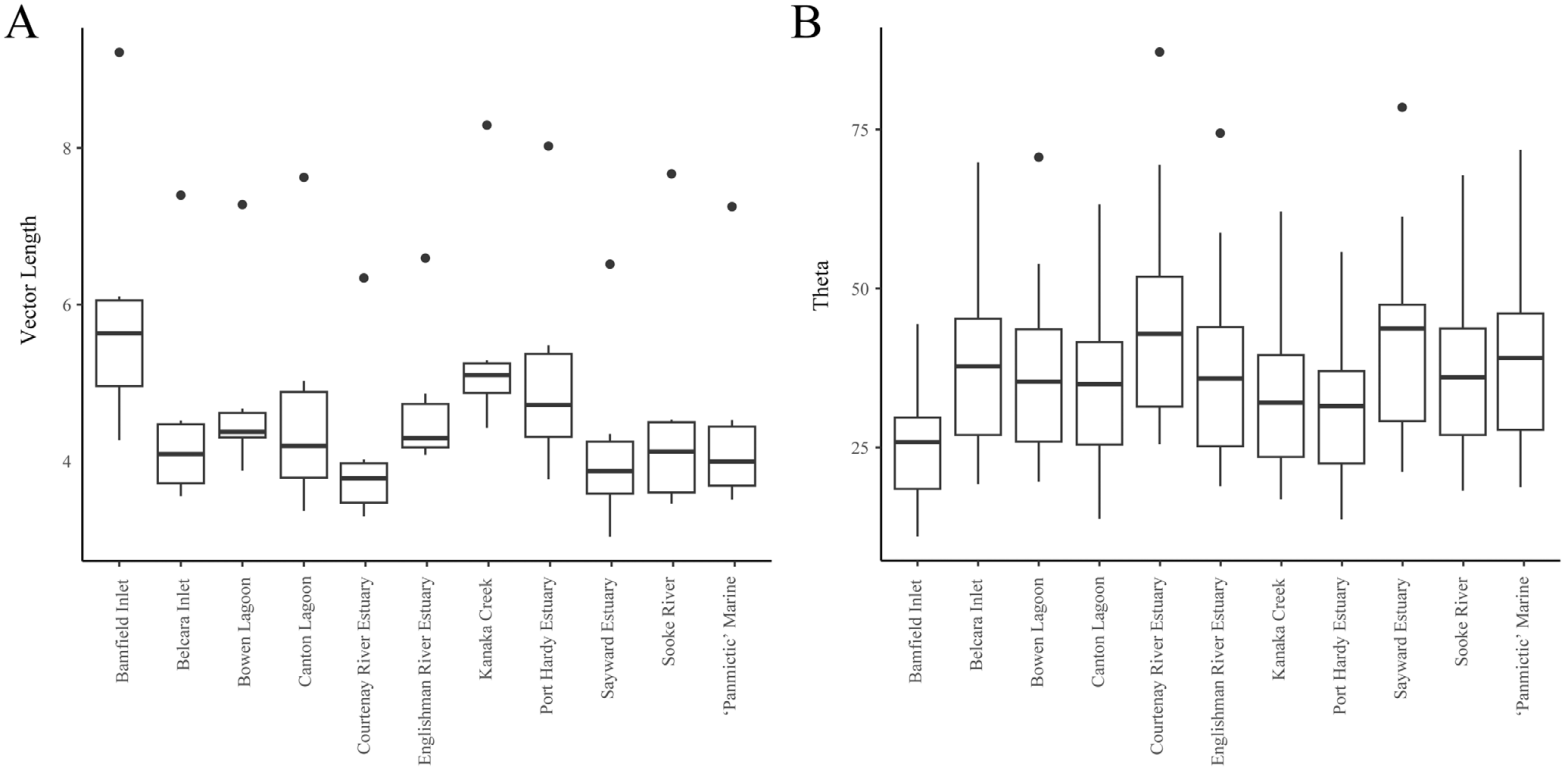
Effect of marine reference on estimates of vector length and parallelism for the six focal freshwater populations. (A) Multivariate vector length, an estimate of morphological divergence. (B) Theta, an estimate of the magnitude of parallelism between pairs of freshwater populations. Ten independent marine sampling locations, along with a panmictic marine reference, are given along the *x*‐axis. The points plotted outside of the interquartile range and whisker reflect outliers.

Multivariate vectors were drawn between all marine and freshwater populations. The magnitude of freshwater divergence was estimated based on the total length of these vectors. Vector length also significantly depended on the marine reference (Marine SS = 15.78, *F*_9_ = 15.5, *p* < 0.0001) (Figure 7A). The variance for freshwater site (random effect) was 1.6 (SE 1.3), indicating moderate variation in this pattern among lakes. The angle (theta) was estimated between all pairs of marine‐freshwater vectors to determine whether there was evidence of parallel evolution. The angle (theta) between pairs of marine‐freshwater vectors was on average 36° (range 11°–87° across the dataset), significantly less than the null expectation of 90° (Stuart et al. 2017) and consistent with a pattern of parallel evolution. Yet, mean theta ranged from 25° to 42° depending on the marine reference used (Figure 7B). In fact, theta significantly differed depending on the marine site used as the shared reference between the freshwater sites (Marine SS = 5081.9, *F*_9_ = 42.0, *p* < 0.0001). The variance for freshwater site (random effect) was 162.2 (SE 12.67), indicating considerable variation in the pattern among lakes. Theta was not significantly related to the average geographic distance of the marine and freshwater sites being compared (*β* = −5.4, SE = 2.7, *t* = −1.99, *p* = 0.06). We find that use of a ‘panmictic’ marine reference, where samples are pooled across all marine sites, resulted in intermediate estimates of freshwater divergence and parallelism (Figure 7).

## 4 Discussion

Relatively limited surveys of marine stickleback have led to the assumption that marine stickleback are morphologically invariant. Yet, phenotypic homogeneity of marines is somewhat counter to the known occurrence of standing genetic variation among marine fish (Barrett et al. 2008; Jones et al. 2012; Feulner et al. 2013) and demonstrated genetic differentiation across marine sampling sites (DeFaveri et al. 2013; Morris et al. 2018). In this study, using samples collected at a relatively small spatial scale, we find evidence of morphological diversity in marine stickleback. For several traits, there was more phenotypic variability among freshwater sites than marine sites, which is consistent with prior work; high degrees of phenotypic variation among freshwater populations are a classic feature of the stickleback adaptive radiation (Bell and Foster 1994). Previous European surveys have also shown greater divergence among freshwater populations relative to marine (Leinonen et al. 2006). Yet, for several traits, we found evidence of considerable phenotypic variation both within and across marine sampling sites. Indeed, for these traits, the level of variation in marine fish was very similar to, or exceeding, that seen in freshwater populations.

Given the level of observed phenotypic variation among marine collection sites we sought to determine whether this variation affected the inferred pattern of freshwater evolution. To do this we used different marine sites as the point of reference for estimating the magnitude of freshwater phenotypic divergence, with the marine fish acting as a proxy for the ‘ancestral state’, as is often done in this system. We found that the magnitude of freshwater divergence, as estimated as the difference of PCA axis 1 eigen values or multivariate vector length, depended on the marine site used as a reference. The effect of marine reference could be quite large, potentially resulting in a 65% greater estimate of freshwater divergence. Consistent with patterns of ecological distance decay (Soininen et al. 2007), we found that the divergence estimates depended on the geographic and environmental distance between the marine and freshwater site, increasing with distance between sites. Interestingly, between marine sites there was no evidence of morphological divergence scaling with geographic distance or ecological similarity. Since our environmental data were limited to climatic factors it may be that other ecological factors such as salinity, which varies greatly in this region, is more important in structuring phenotypic variation in marine habitats. Furthermore, it remains to be determined whether there is fine scale population structure that corresponds to the phenotypic variation.

The data presented here suggest that care should be taken when selecting marine sites to use as a proxy for the ancestral state in estimations of freshwater divergence. The effect of reference choice has previously been noted in population genetic studies. For example, in westslope cutthroat trout, estimates of ancestry quotient significantly depended on the genetic background of the reference population of rainbow trout, which varied from wild native populations to introduced or hatchery lines (Allen et al. 2016). Reassuringly, in this stickleback example, we found that the rank of divergence estimates was maintained across freshwater populations, indicating that relative comparisons of divergence for freshwater populations against a common reference would be unaffected by marine reference choice. However, this pattern may not hold at larger spatial scales, as there was a relatively small spatial scale of sampling in our analysis—distances ranged from 10 to 221 km between freshwater sites and 10–284 km between paired marine and freshwater sites. Our data suggest that when surveying multiple freshwater populations from a region, it would generally be most appropriate to generate estimates of phenotypic and genotypic divergence using marine fish collected at a single site that is geographically close to the freshwater sites. If freshwater sampling sites are spread over larger geographic scales (> 500 km) it may be worth considering surveying multiple marine sites and either pooling the marines across sites to generate a ‘panmictic’ marine reference pool (as demonstrated in Figures 5 and 7) or repeating estimates using different marine sources to see how estimates are affected by the ancestral proxy to assess robustness.

Threespine stickleback are a prominent example of parallel evolution, with many phenotypes (Conte et al. 2012; Stuart et al. 2017) and their underlying genotypes (Colosimo et al. 2005; Jones et al. 2012; Rennison et al. 2019, 2020) exhibiting a pattern of repeatability across independent freshwater populations adapting to similar ecological conditions. Across the replicate freshwater populations surveyed in this study, we find strong evidence of parallelism; for all pairwise comparisons, the angle (theta) estimated between the divergence vectors of marine‐freshwater population pairs was much smaller than the null expectation of 90° (Stuart et al. 2017). Yet, theta differed significantly depending on the marine site used as a reference. Substitution of the marine reference resulted in up to a two‐fold difference in theta. For future investigations of phenotypic parallelism in freshwater populations, it will be important to consider that the strength of observed parallelism is dependent on the marine proxy used. Unfortunately, for parallelism analyses, it is more difficult to make a recommendation on the geographic sampling strategy. This is because there was no significant relationship between theta and geographic distance. Thus, we recommend estimation of theta for all pairwise freshwater sites using more than one marine reference. This would allow researchers to determine whether the inferred pattern of parallelism is robust to the choice of marine proxy.

## Author Contributions

**Brandon Tsai:** conceptualization (equal), data curation (lead), formal analysis (lead). **Elizabeth Tapanes:** conceptualization (supporting), data curation (supporting), formal analysis (supporting), methodology (supporting). **Ainsley L. Fraser:** resources (supporting). **Rana El‐Sabaawi:** project administration (supporting), resources (supporting). **Diana J. Rennison:** conceptualization (lead), data curation (equal), formal analysis (equal), funding acquisition (lead), investigation (lead), methodology (equal), project administration (equal), resources (lead), supervision (lead), writing – original draft (equal), writing – review and editing (lead).

## Conflicts of Interest

The authors declare no conflicts of interest.

## Data Availability

Data and underlying code are archived on GitHub (link: https://github.com/djrennison/Marine_Freshwater_Divergence/tree/main).
